# Harnessing artificial intelligence to reduce phototoxicity in live imaging

**DOI:** 10.1242/jcs.261545

**Published:** 2024-02-07

**Authors:** Estibaliz Gómez-de-Mariscal, Mario Del Rosario, Joanna W. Pylvänäinen, Guillaume Jacquemet, Ricardo Henriques

**Affiliations:** ^1^ Instituto Gulbenkian de Ciência, Oeiras 2780-156, Portugal; ^2^ Faculty of Science and Engineering, Cell Biology, Åbo Akademi University, Turku 20500, Finland; ^3^ Turku Bioscience Centre, University of Turku and Åbo Akademi University, Turku 20520, Finland; ^4^ Turku Bioimaging, University of Turku and Åbo Akademi University, Turku 20520, Finland; ^5^ InFLAMES Research Flagship Center, Åbo Akademi University, Turku 20100, Finland; ^6^ UCL Laboratory for Molecular Cell Biology, University College London, London WC1E 6BT, UK

**Keywords:** Photodamage, Phototoxicity, Live-microscopy, Artificial intelligence, Deep learning, Data-driven microscopy, Fluorescence microscopy, Live-cell super-resolution microscopy

## Abstract

Fluorescence microscopy is essential for studying living cells, tissues and organisms. However, the fluorescent light that switches on fluorescent molecules also harms the samples, jeopardizing the validity of results – particularly in techniques such as super-resolution microscopy, which demands extended illumination. Artificial intelligence (AI)-enabled software capable of denoising, image restoration, temporal interpolation or cross-modal style transfer has great potential to rescue live imaging data and limit photodamage. Yet we believe the focus should be on maintaining light-induced damage at levels that preserve natural cell behaviour. In this Opinion piece, we argue that a shift in role for AIs is needed – AI should be used to extract rich insights from gentle imaging rather than recover compromised data from harsh illumination. Although AI can enhance imaging, our ultimate goal should be to uncover biological truths, not just retrieve data. It is essential to prioritize minimizing photodamage over merely pushing technical limits. Our approach is aimed towards gentle acquisition and observation of undisturbed living systems, aligning with the essence of live-cell fluorescence microscopy.

## Teaser

**Summary:** When conducting live-cell imaging, it is important to balance sample health with image quality. AI technology can help achieve this balance, allowing for reactive and gentle imaging.

## Introduction

The ability to comprehend biological events is inherently linked to the capacity for non-invasive observation. Fluorescence microscopy has been instrumental in facilitating these analyses across a range of scales (Heimstädt, 1911; Lehmann, 1913; Reichert, 1911). Over the past two decades, technological advancements, such as light-sheet microscopy (Dodt et al., 2007; Huisken et al., 2004; Reynaud et al., 2008; Verveer et al., 2007), structured illumination microscopy (SIM) (Gustafsson, 2000; Heintzmann and Huser, 2017) and single-molecule localisation microscopy (SMLM) (Betzig et al., 2006; Hess et al., 2006; Lelek et al., 2021), have revolutionised fluorescence light microscopy, enabling us to characterise biological events from molecular interactions up to larger living organisms.

Advanced microscopy imaging generally needs high levels of fluorescence excitation light, which results in phototoxicity or photodamage. These terms refer to the detrimental impacts of light, especially when employing photosensitising agents or high-intensity illumination, and are a key challenge for live microscopy imaging (see https://focalplane.biologists.com/2021/05/14/phototoxicity-the-good-the-bad-and-the-quantified/ and Reiche et al., 2022; Tinevez et al., 2012; Wäldchen et al., 2015). Although toxicity is only an issue for living systems, photodamage also occurs in non-living materials and thus, for simplicity, both terms are used here interchangeably. Sample illumination might also result in photobleaching, a process characterised by an irreversible loss of a fluorescent signal attributed to the destruction of the fluorophore. This is one manifestation of light damage, among other possible effects. Phototoxicity severely influences the experimental outcomes by altering biological processes under observation, skewing findings and impeding consistency (Alghamdi et al., 2021). Therefore, it is crucial during live-cell microscopy to carefully consider these factors to prolong the duration of imaging and achieve dependable research outcomes (Icha et al., 2017; Kiepas et al., 2020; Laissue et al., 2017; Mubaid and Brown, 2017; Tosheva et al., 2020).

**Fig. 1.**
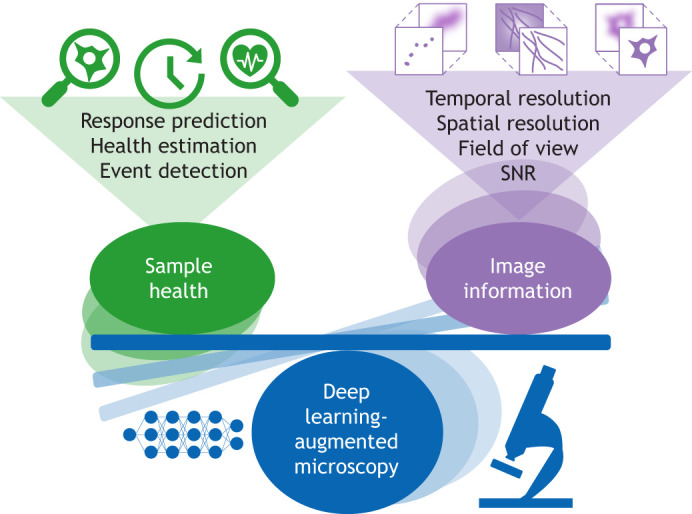
**Integrating deep learning with live-cell microscopy.** The delicate balance between sample health and the information obtained by imaging requires a compromise between both elements. Deep learning-augmented microscopy aims to reduce this compromise, striving to obtain equal information from our sample with less impact on its health.

The biological validity of live-cell imaging experiments requires a precise balance between acquiring high quality data that can be analysed and maintaining the health of the specimen (depicted in Fig. 1). Major advancements have been made in both hardware and software technologies, aiming to reduce light damage of the sample. Importantly, super-resolution techniques, such as stimulated emission depletion (STED), achieves nanoscale spatial resolution by eliminating the diffraction barrier, at the cost of damaging the sample due to the high illumination intensity required (Hell and Wichmann, 1994). Reversible saturable optical fluorescence transition (RESOLFT) overcomes the limitation of STED, that is the high degree of photobleaching and photodamage of the sample, as it requires much lower light intensities that are comparable to those used in confocal microscopy (Hofmann et al., 2005; Ratz et al., 2015) (Table 1). Hardware innovations, such as lattice light sheet (LLS) microscopy (Chen et al., 2014) and Airyscan microscopy (Huff, 2015) are notable examples of gentler acquisition approaches for the sample health that still accomplish high resolutions (Table 1). Additionally, computational advancements such as fluctuation-based super resolution microscopy offer promising solutions to photodamage (Dertinger et al., 2009; Gustafsson et al., 2016; Laine et al., 2023). A recent study has shown that a two-colour illumination scheme combining near-infrared illumination with fluorescence excitation has the capacity to limit the phototoxicity caused by light-induced interactions with fluorescent proteins (Ludvikova et al., 2023). These technological breakthroughs have the potential to optimise observation accuracy while mitigating photodamage.

**Table 1.**
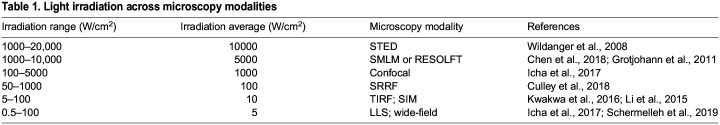
Light irradiation across microscopy modalities

In parallel, artificial intelligence (AI), specifically deep learning, can significantly improve imaging information and analysis in low-illumination scenarios by considerably enhancing image quality and quantification (Belthangady and Royer, 2019; Melanthota et al., 2022; Tian et al., 2021). This has inspired the search for integrated solutions by the microscopy community (Bouchard et al., 2023; Ebrahimi et al., 2023; McAleer et al., 2021; https://www.microscope.healthcare.nikon.com/en_EU/resources/application-notes/reduction-of-phototoxicity-of-fluorescent-images; Wagner et al., 2021). The fusion of advanced optical hardware with computational models and AI heralds new breakthroughs in overcoming the sample damage that is induced by traditional live fluorescence microscopy methodologies, marking the advent of AI-enhanced smart microscopy (Fig. 1).

In this Opinion piece, we will first examine the mechanisms of phototoxicity and strategies for its quantification. Next, we delve into how deep learning can enhance microscopy image analysis, while supporting more sample-friendly imaging setups. Finally, we explore smart microscopes that integrate deep learning to balance sample health and data quality in real-time acquisitions (Fig. 1). Throughout, we aim to make the case that, although computational advances are powerful, we must ensure that biological relevance is the central focus. As AI continues to enhance imaging capabilities, we must maintain sight of the overarching goal – to uncover biological truths without or with only minimal disturbance. Rather than blindly pushing the physical limits of microscopy, future AI-enabled technologies should be designed to extract maximal information through minimal invasiveness. Universal standard metrics of photodamage would aid this pursuit, enabling quantitative assessments of imaging protocols. We argue that embracing this balanced perspective is crucial for developing microscopes that truly observe life with minimal perturbance. Our aim is to emphasize that striking the right equilibrium between sample health and data quality will allow AI to fully realise the promise of gentle yet highly informative live-cell fluorescence microscopy.

## Phototoxicity quantification

Fluorescence microscopy uses fluorescent reporters to visualize cell components and activities (Heimstädt, 1911; Lehmann, 1913). However, exciting fluorophores with light inevitably enhances the generation of reactive oxygen species (ROS) through interactions with ambient oxygen. At physiological levels, ROS participate in signalling and are present in regular cellular processes. However, excessive ROS result in oxidative stress and perturb the biological processes under observation – an effect termed phototoxicity or photodamage when these are caused by light (Icha et al., 2017; Laissue, 2021; Reiche et al., 2022).

The primary ROS-related molecules include hydroxyl radicals, hydrogen peroxide, nitric oxide and singlet oxygen, which readily oxidize biomolecules, such as lipids, proteins and DNA (Eichler et al., 2005; Hockberger et al., 1999). Higher-intensity UV and blue excitation light can also directly damage DNA by producing thymine dimers (Zhang et al., 2022b). Additionally, fluorophores photobleach via ROS generation upon light exposure (Demchenko, 2020). Although interrelated, photobleaching and photodamage are distinct and can occur independently (Ludvikova et al., 2023).

At the cellular level, accumulating oxidative stress disrupts redox homeostasis and normal physiology (Icha et al., 2017; Tosheva et al., 2020). Effects span mitochondrial fragmentation, cytoskeletal derangements, stalled proliferation and loss of motility (Alam et al., 2022; McDonald et al., 2012; Zhang et al., 2022b). In whole organisms, this manifests as tissue degeneration, developmental defects and apoptosis (Laissue et al., 2017).

Considering the varying light energy requirements current microscopy modalities employ, many preventive strategies exist to reduce the effects of phototoxicity, such as limiting light irradiation by reducing the acquisition points or the light dose (Kiepas et al., 2020; Mubaid and Brown, 2017; Reynaud et al., 2008), using light detectors as an array of 32 GaAsP-PMT detectors or highly sensitive sCMOS cameras (Huff, 2015; Saxena et al., 2015) and performing bioluminescence-based assays that reduce the amount of light required (Suzuki et al., 2016). Other strategies focus on controlling oxidative stress effects in biological samples by supplementing antioxidants (Harada et al., 2022 preprint; Kesari et al., 2020) or chemically increasing the oxidative stress resistance of the sample itself (Kunkel et al., 2018). Unfortunately, the degree of photodamage elicited varies based on multiple factors, including sample traits, illumination parameters and imaging modality (Table 1) (Laissue et al., 2017; Reiche et al., 2022; Tinevez et al., 2012). For example, actively dividing cells better tolerate photodamage than post-mitotic neurons (Stevenson et al., 2006). Additionally, the imaging modality is particularly critical for sample health, as techniques that yield a higher signal-to-noise ratio (SNR) such as super-resolution methods [STED, SMLM, SIM, total internal reflection fluorescence (TIRF) and confocal microscopy], generally require higher light energy than low SNR or standard-resolution methods (LLS or wide-field microscopy), creating a higher negative impact on the sample (Table 1) (Betzig et al., 2006; Blom and Brismar, 2014; Dertinger et al., 2009; Dodt et al., 2007; Gustafsson, 2000; Gustafsson et al., 2016; Heintzmann and Huser, 2017; Hess et al., 2006; Huff, 2015; Klar and Hell, 1999; Lelek et al., 2021). For this reason, designing a strategy to prevent photodamage remains challenging. Namely, there are no universal live-cell imaging metrics that can relate to the light exposure and consequent damages of the sample and that can be used to assess and optimise imaging systems. Quantifying photodamage can be used to tune the acquisition parameters to reduce the fluorescence light illumination and establish a doable compromise between the sample health and, accordingly, image information (Fig. 1). Excitingly, a quantitative measurement of phototoxicity can be used to train a smart virtual component that decides automatically towards less aggressive image acquisition parameters and triggers these accordingly in the microscope. Importantly, such universal metrics would also support the reproducibility of biological readouts and improve result robustness. By contrast, without these universal metrics, it is challenging to fully leverage the capacity to image biological systems since we are not assessing potential damage that arises from the imaging itself. This impedes the assessment of experimental conditions to achieve maximum spatial and temporal resolution while preserving cell viability (Box 1).

Box 1. The relationship between fluorescence excitation light, image information and phototoxicity Modern microscopy methods aim to minimise required illumination by targeting specific information for visualisation. However, acquired image quality depends on several factors, including the SNR, contrast and spatiotemporal resolution. Each microscopy technique has inherent limitations that constrain optimising these properties. This necessitates balancing trade-offs between them, described as the ‘microscope pyramid of frustration’ (Scherf and Huisken, 2015; Weigert et al., 2018). For super-resolution microscopy, this trade-off space was recently characterised (Jacquemet et al., 2020; Tosheva et al., 2020). The balance can be tuned to experimental needs by adjusting light exposure and acquisition speed – both common levers to enable gentler imaging. As such, methods such as deep learning that computationally enhance image quality from minimally invasive acquisitions are particularly valuable. They alleviate the trade-off between image information and phototoxicity constraints (Fig. 1). Indeed, the growing capacity of deep learning to refine image-based information is attracting interest from the microscopy community. It is becoming a popular strategy to enable reduced phototoxicity imaging setups (Ebrahimi et al., 2023; Scherf and Huisken, 2015).

Numerous known markers exist to identify and characterise sample damage based on the previously mentioned phototoxicity hallmarks (Alghamdi et al., 2021; Laissue et al., 2017). However, most of these phototoxicity markers require the use of fluorescence or luminescence light excitation to report back information. Thus, incorporating them might compromise fluorescent channels usually reserved for observing conditions of interest (e.g. markers for DNA oxidative damage) and, when paired with live-cell experiments, could increase the phototoxicity risk on the specimen due to the interaction of light with oxygen radicals. Yet, quantification-based screenings of phototoxicity are less commonly employed than the observation and experience of the researchers to to assess cell health (Laissue et al., 2017; Tosheva et al., 2020; Wäldchen et al., 2015). Although there are some label-free attempts to provide quantifiable metrics for the assessment of imaging setups and support improving sample viability, they often simplify the impact of fluorescence excitation light to a binary classification of viable/healthy or non-viable/dead (Icha et al., 2017; Richmond et al., 2017 preprint; Tinevez et al., 2012; Wäldchen et al., 2015). By considering the decline of cell health and recovery as valid photodamage stages for an image-based classifier, the assessment of phototoxicity could be more flexible. Here, a gradient model that considers the accumulation of discrete minor effects would more accurately depict the spectrum of effects, as documented in the existing literature. For example, the heartrate in zebrafish embryo development was recently used as a gradual quantitative measure of phototoxicity and used to optimise a multiphoton light-sheet microscopy acquisition set up (Maioli et al., 2020). The ability of deep learning to extract meaningful and general features from big data has enabled image-based cell profiling, phenotyping and even encoding of metastatic potential. One could for example, think about using equivalent techniques to identify, encode and model photodamage based on imaged cell morphology or monitored cell behaviour (Caicedo et al., 2017; Chandrasekaran et al., 2021; Doron et al., 2023 preprint; Wu et al., 2020). Despite these advances in image analysis, general metrics based on cell physiological cues to assess photodamage in live-cell imaging across different biological samples and imaging setups are missing.

As advanced image analysis tools become increasingly available, a future strategy could incorporate specific phototoxicity assessment within the automated image acquisition and analysis workflows. We would suggest less aggressive but still sufficiently accurate imaging approaches in terms of resolution and SNR, such as holotomography microscopy, to monitor sample health. These types of automated observations, paired with standardised experimental guidelines for identifying and quantifying phototoxic events, represent a promising solution. We, therefore, argue that adopting such methods should be prioritised by scientists aiming to create robust imaging strategies for visualising biological phenomena.

## Deep learning for microscopy to the rescue

**Fig. 2.**
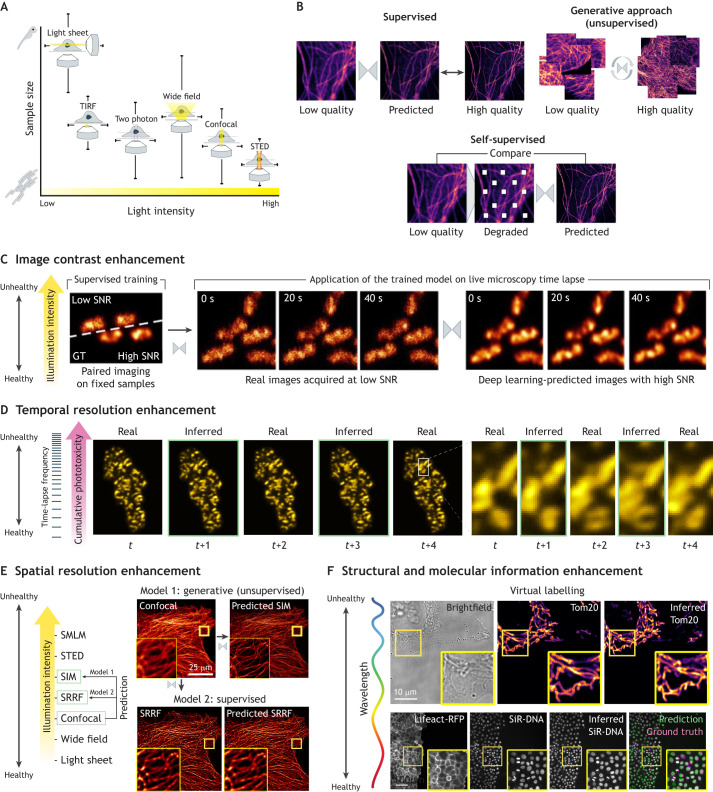
**The deep learning landscape for a gentler live-cell microscopy imaging.** (A) Comparison of the light intensities of different light microscopy modalities on a sample according to its size. Some modalities such as light-sheet microscopy use a lower amount of light on the sample to increase cell survival. This modality is commonly used to image embryos owing to its reduced phototoxicity, but it provides lower spatial resolution. Other modalities, such as STED sacrifice sample health to gain spatial resolution, as they require high light intensities. Typically, the microscopy imaging setups designed for nanoscale resolution are used on microorganisms, such as cell cultures, and necessitate higher laser powers and are more aggressive for the living matter being imaged. (B) Common approaches to train deep learning methods in the specific context of image denoising (see Box 2). Supervised training requires datasets of paired images so given an input noisy image (low quality), the output of the network (inferred image) is compared with the expected ground truth image (high quality) to compute a loss value and train the network. In generative approaches, the images are not paired so the network learns the distribution of the low quality and high-quality image datasets and how to translate one into the other one. The grey arrowheads pointing at each other represent the cycle in unsupervised generative approaches of translating an image from one distribution into the other one and the translation back using a neural network. Self-supervised approaches are used when only a dataset of low-quality images is available. Here, the input image is transformed to virtually create a paired image that represents a pseudo-ground-truth and can be used to train the network. (C–F) Deep learning-augmented microscopy. Deep learning models can be used to enable microscopy acquisitions that use lower fluorescence light intensities or illuminate the sample less often. Thereafter, the images are processed with a model trained for a specific task. (C) Image contrast enhancement with denoising and restoration. High illumination intensities are used to obtain images with a high signal-to-noise-ratio (SNR) at the expense of causing photobleaching, among other effects that can be detrimental to the sample. Reducing the fluorescence illumination intensity prevents photobleaching but results in images with a low SNR. By using deep learning, the acquisition duration can be extended by lowering the laser power and acquiring images with a low SNR and decreased photobleaching. An image restoration model can be trained on pairs of images from fixed samples, which facilitates creating perfectly aligned pairs of low and high SNR. After training and evaluation, the model can be used to process the more gently acquired low SNR time-lapse movies and enhance contrast, recovering the image quality of high illumination setups. GT, ground truth. (D) Temporal interpolation. Here, the temporal resolution is improved by training a model to predict the intermediate time points between two given frames. (E) Spatial resolution enhancement. A super-resolution model is trained to translate images from one modality (e.g. confocal) into another one (e.g. SIM or SRRF). Depending on the availability of paired images for the training, one should choose between a generative or a supervised learning approach. (F) Structural and molecular information enhancement. The phototoxic effects of light are not equal across wavelengths. Longer wavelengths, such as red light, are less phototoxic than shorter ones, such as UV light. Given that it is not always possible to use less damaging light wavelengths options, one can opt to circumvent the partial use of fluorescence illumination by using virtual labelling approaches that can generate labelling out of existing structures from bright-field, autofluorescence or crosstalk between channels. Images in B, E and F were extracted and modified from von Chamier et al. (2021) and those in C and D from Spahn et al. (2022) which were both published under an CC-BY 4.0 license.

The recent advancements in deep learning have laid a solid foundation for the growing field of deep learning-augmented microscopy (Pylvänäinen et al., 2023), which holds great promise due to the flexibility it introduces for imaging experiments (Belthangady and Royer, 2019; Meijering, 2020; Melanthota et al., 2022; Moen et al., 2019; Tian et al., 2021). Among all the existing techniques for microscopy image processing, many possibilities exist to reduce phototoxicity (Fig. 2). Previous discussions (Tian et al., 2021) divide such techniques between strategies that aim either to surmount the physical limitations intrinsic to live fluorescence microscopy imaging (i.e. acquisition speed or illumination) or to enhance the content in qualitatively less superior but more sample-friendly image data (Box 2). The former includes techniques, such as denoising, restoration or temporal interpolation, which allow reduced light exposure by using lower laser powers or lower acquisition frame rate. The latter, referred to by the original authors as ‘augmentation of microscopy data contrast’, includes techniques such as virtual super-resolution (Chen et al., 2021; Jin et al., 2020; Qiao et al., 2021, 2022; Wang et al., 2019; Zhang et al., 2022a).

Box 2. Guidelines for annotation and model training to optimize deep learning for microscopy image analysis Traditionally, deep learning models are trained in a supervised – using paired input-output image datasets – or unsupervised – the model learns patterns and insights from unlabelled input images without any explicit guidance – manner (Fig. 2B). Supervised approaches have demonstrated superior accuracy and specificity to the task and data distribution, but their versatility requires the availability of paired images. Microscopy imaging allows for creating paired image datasets by alternating acquisition setups (e.g. channels) and combining different modalities (e.g. paired widefield microscopy and SIM) (Qiao et al., 2021; von Chamier et al., 2021), simulating data (Fang et al., 2021; Nehme et al., 2018; Oh and Jeong, 2023; Sage et al., 2019; Saguy et al., 2023) or, recently, by developing correlative approaches, such as correlative light and electron microscopy (CLEM) (de Boer et al., 2015). However, cases remain in which the obtaining of paired input and output images to train deep learning models is still a limitation. For example, in live imaging, paired acquisitions can be complicated by sample movements or photobleaching. Alternatively, one could acquire paired images of *ex vivo* samples – providing perfectly aligned images for training and assessment – to subsequently perform inference with *in vivo* images (Fig. 2C) (Spahn et al., 2022; Weigert et al., 2018; Xu et al., 2023). Importantly, collecting images from fixed samples supports the faster creation of more extensive and diverse datasets than live imaging. However, there are scenarios in which such paired datasets do not encapsulate the complexity of live experiments, are not experimentally feasible or where cross-modality acquisition devices are inaccessible. Therefore, this limitation, as well as time-consuming data annotation processes, has propelled the exploration into alternative approaches, such as semi- or weakly supervised (Bilodeau et al., 2022), self-supervised (Krull et al., 2019, 2020) or generative techniques (Li et al., 2022a; Wang et al., 2019; Xu et al., 2023) (Fig. 2B).

Recent advances in denoising and restoration using deep learning have shown promising capabilities to support live-imaging setups with reduced phototoxicity. For example, these methods can virtually remove noise, enhance SNR and improve fluorescence channel contrast in images acquired under low illumination conditions (Krull et al., 2019, 2020; Weigert et al., 2018; Zhang et al., 2022a) (Fig. 2C). Other techniques can computationally reconstruct isotropic 3D volumetric information from sparse optical sectioning data (Chen et al., 2021; Guo et al., 2020; Li et al., 2023, 2022b; McAleer et al., 2021; Park et al., 2022). Such capabilities allow microscopists to use gentle imaging protocols with reduced fluorescence excitation or sparse *Z*-stack sampling, while still recovering high-quality image data computationally after acquisition. Specifically, deep learning models can be trained on paired datasets from low- and high-illumination imaging of the same samples (Box 2). The models then learn to enhance contrast and virtually recover the lost information when applied to new low-exposure test data. This strategy to reduce phototoxicity is also being adopted by commercial solutions such as Enhance.ai, currently part of the Nikon NIS-Elements imaging software (https://www.microscope.healthcare.nikon.com/products/software/nis-elements). These approaches are inherently gentler on live specimens because they support imaging setups with reduced phototoxic excitation levels. Similarly, intelligent temporal interpolation techniques, such as content-aware frame interpolator (CAFI) or DBlink, allow for slowing down acquisition frame rates, while accurately reconstructing missing timepoints later using deep learning (Priessner et al., 2021 preprint; Saguy et al., 2023) (Fig. 2D). As discussed above, reducing the number of illumination timepoints can substantially decrease cumulative photodamage. Longer intervals between acquisitions potentially enables biological recovery processes to repair photodamage, further supporting longer term live imaging. However, one should be cautious when using these techniques for further quantifications other than segmentation and tracking, such as intensity-based quantifications (further discussed below).

Another innovative approach to enable reduced illumination imaging setups is exploiting cross-modal style transfer methodologies. In brief, these methods involve training a deep learning model to computationally convert the style of an image to mimic that from a different imaging modality (Fig. 2F). For example, it has been shown that SIM images can be inferred from input images that have been acquired with wide-field illumination, which reduces the photon dose by a factor of 9 in 2D and 15 in 3D (Qiao et al., 2021). This capability extends to numerous types of fluorescence microscopy modalities, such as confocal to STED (Bouchard et al., 2023; Wang et al., 2019), SIM and super-resolution radial fluctuations (SRRF) microscopy (von Chamier et al., 2021), or wide-field to SMLM (Macke et al., 2021; Nehme et al., 2018; Ouyang et al., 2018). The enhancement in spatial resolution through learning of the fine details of a sample is similar in objective to traditional deconvolution. It offers comparable benefits for mitigating phototoxicity, as it allows to generate enhanced resolution data from gentler imaging methods (widefield and/or confocal against SIM, STED and SMLM) (Table 1). These techniques have some limitations that are subject to debate. For instance, they might have limited accuracy when predicting fluorescence intensity, which can make it challenging to measure protein stoichiometry. However, their ability to enhance image quality has a direct effect on subsequent tasks like localization, tracking and segmentation, thereby improving their accuracy (Belthangady and Royer, 2019; Hutson, 2018; Priessner et al., 2021 preprint; Weigert et al., 2018). It has been suggested that less aggressive live imaging approaches might cause aesthetical blur and be less appealing. However, such datasets can comprise easier-to-interpret data due to the reduction in the biological artefacts that are induced by photodamage (e.g. apoptosis, stressed cells or specimen shrinkage during illumination) and have the benefit of preserving close-to physiological conditions (Weigert et al., 2018).

By exploiting the capability of data-driven methods, virtual (or artificial) labelling approaches have emerged (Fig. 2F). Virtual labelling uses deep learning to computationally predict fluorescence labelling patterns and signals directly from an image, representing yet another fluorescence channel or from transmitted light images, without actual fluorescent tags. By inferring some of the biological structures through deep learning methods, virtual labelling allows end users to eliminate the most harmful illumination wavelengths from their experiments. For example, cell nuclei (e.g. Hoechst staining excited with an ∼475 nm laser) can be virtually inferred from actin (e.g. Lifeact staining excited with an∼561 nm laser), which results in a halved light dose compared with an imaging setup that illuminates both channels (von Chamier et al., 2021). Likewise, artificial labelling can be employed for spectral unmixing, which offers several key advantages, including illumination channel reduction and acceleration of image acquisition (Fig. 2F) (Jiang et al., 2023 preprint; McRae et al., 2019; Xue et al., 2022). One could also eliminate the need for sample exposure to excitation light by estimating specific fluorescence information (e.g. nucleoli, cell membrane, nuclear envelope, mitochondria or neuron-specific tubulin) from brightfield input images (Christiansen et al., 2018; Ounkomol et al., 2018; von Chamier et al., 2021). It is worth noting that the latter technique is also categorized as a cross-modality style transfer approach. All these approaches enable the acquisition of images with less explicit information that, after virtual labelling, can be quantitatively processed as if the fluorescence information had been acquired. Among these benefits, the former is pivotal in enabling more sample-friendly setups, and indeed, virtual labelling is often suitable as an intermediary step for further quantification, such as segmentation or tracking (Hollandi et al., 2020; von Chamier et al., 2021).

Of note, most cited approaches here can now be widely adopted thanks to the different software developments that enable the training, deployment and sharing of deep learning models in a user-friendly manner (Gómez-de-Mariscal et al., 2021; Ouyang et al., 2022 preprint; Spahn et al., 2022; von Chamier et al., 2021). We expect that with the growth of these resources along with a more easily accessible high performing computational power, the deep learning augmented microscopy will be more widely harnessed in image driven life-sciences research.

## Gentle smart microscopes

**Fig. 3.**
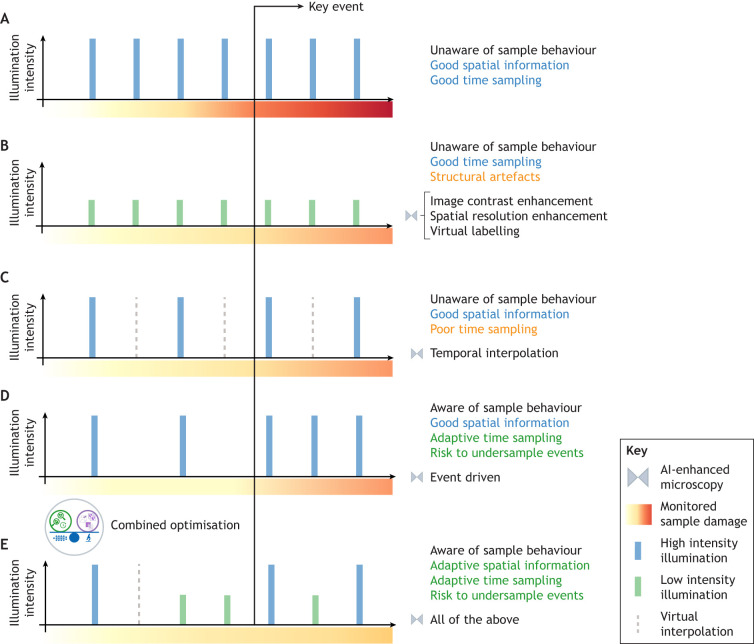
**Prospective imaging with AI-enhanced live-cell microscopy.** Shown here are examples of different acquisition frequencies and illumination intensities in a live-cell microscopy imaging experiment that is used to observe a particular key event. The gradient bar represents the estimated health sample damage during the acquisition. The length of the acquisition steps represents high (long blue bars) and low (short green bars) illumination intensities. (A) Uniform fast time-frequency with high illumination intensities allows for high spatial and temporal resolution at the expense of drastically damaging the sample and observing a key event under unhealthy conditions. (B) Uniform fast time-frequency with low illumination intensities allows for a gentle acquisition with high temporal resolution but with low SNRs or suboptimal spatial resolution. Using the deep learning approaches shown in Fig. 2, the image quality could be improved at the risk of generating structural artefacts. (C) Uniform slow time-frequency with high illumination intensities allows for high spatial resolution in a gentler manner but critical information about the event of interest might be missed. A deep learning-based temporal interpolator could partly recover the information obtained from the set-up in A. (D) Non-uniform event-driven acquisition in which the high-intensity illumination is triggered by the automatic identification of specific hallmarks in the field of view. This approach allows for a gentler adaptive sampling with good structural and temporal resolution, but can be biased towards the data used to train or codify event identification. (E) A combined optimisation of the above approaches that balances the health state of the imaged sample and the quality of the information at specific time points. The system uses image contrast enhancement or temporal interpolations to improve image quality during non-event acquisition, allowing a gentler acquisition. When an event of interest is anticipated, it automatically speeds up the acquisition and decides on appropriate illumination intensities. This preserves the information needed about the event of interest without drastically increasing the induced phototoxicity on the sample.

The integration of AI components directly into the fluorescence microscopy acquisition sequence shows great promise for minimizing photodamage in real-time and enabling accurate observations of biological dynamics. Analogous to autonomous vehicles or intelligent industrial robots, microscopes can incorporate AI capabilities to make real-time decisions by analysing the observed image data and integrating them into an intelligent feedback loop. This loop is responsible for analysing the data that is being observed in real time and updating the imaging parameters (e.g. time-lapse frequency or illumination intensity) based on visual cues; it would balance sample health against image quality to optimize data collection (Figs 1 and 3) (Scherf and Huisken, 2015). For example, in tracking the membrane dynamics of individual cells, the microscope could use a low frame rate to gently image a fluorescent membrane marker, while simultaneously tracking cells via a transmitted light channel at a higher frame rate. This system could identify fast dynamics or ambiguous situations (e.g. two cells moving close together) and balance trade-offs on when and where to increase imaging speed or acquire extra channels, such as nuclear stains, to properly identify each cell. Incorporating quantitative phototoxicity reporter data on cell resilience would further optimize these decisions, as losing a cell track might be preferable to aggressively re-imaging the entire sample. More broadly, developing such smart microscopes is tied to balancing the combination of features (spatiotemporal resolution, SNR, field of view size, fluorescent channels, etc.) that extract the most relevant information against factors that preserve sample health. Likewise, the availability of quantitative metrics for sample health and image information quality, should stimulate the design of AI systems that, after training, would automatically make these decisions driven by the observed data in a smart fashion.

There are already a number of conceptualised and proven approaches towards such gentle smart microscopy. Of those, event-driven approaches automatically identify specific objects or incidents in images acquired in a less phototoxic setup, which triggers their acquisition in real-time (Alvelid et al., 2022; André et al., 2023; Chiron et al., 2022; Fox et al., 2022; Mahecic et al., 2022) (Fig. 3). Although these adaptive approaches reduce the induced phototoxicity by increasing the illumination of the sample when needed, in most cases, they are equipped with deep learning models that are trained to recognise predefined objects or elements in the images. Given its complexity, these might not always be present for biology set-ups, limiting or biasing the observation of novel physiological processes. Alternative approaches propose the integration of image resolution enhancement in the image acquisition loop to obtain faster and gentler setups. For instance, a deep learning model that is trained and validated in the acquisition loop to enhance the volumetric reconstruction of the sample, providing an adaptive light field microscopy (LFM) setup, has been presented (Wagner et al., 2021). In the context of super-resolution imaging, evaluating the quality of virtually inferred STED images from confocal microscopy images has been proposed so that the uncertainty in the observed sample can be determined and a decision on whether a new STED image should be acquired or not made (Bouchard et al., 2023). All these works pose new paradigms in the realm of smart microscopy.

Despite sample health preservation being both a strong motivation and a major limitation in live-cell imaging, none of the currently existing solutions can directly analyse, estimate and integrate information on the sample health into the acquisition loop. Robust photodamage reporters that provide quantitative assessments of sample health without requiring additional fluorescence channels can, therefore, directly contribute to more reproducible biological readouts. This could involve exploring modalities, such as transmitted light microscopy or label-free techniques. Moreover, quantitative reporters could support the design of automated workflows that analyse sample health in real-time during image acquisition rather than only evaluating the image quality (Fig. 3). This would allow the detection of early signs of photodamage and the adaptive determination of optimal imaging conditions. In other words, it will open the door for data-driven sample-oriented live microscopy.

Pursuing such technical innovations while deepening our understanding of the mechanisms that give rise to photodamage will enable microscopists to unlock the full potential of smart imaging. With photodamage-aware AI and automated tools, the goal of observing undisturbed physiological processes can be realised. This will profoundly enhance the capacity of fluorescence microscopy to uncover ground truths in biology.

## Challenges and future outlook

In addition to determining the optimal deep learning approaches for various image-processing tasks, the success of AI-enhanced live microscopy depends on its ability to reliably extract quantifiable physiological information from the acquired image data. Thus, more rigorous validation methodologies and standardized quantitative strategies are still required to ensure both the biological fidelity of computationally restored images and the integrity of recovered signal intensities from techniques that artificially generate or enhance images (i.e. virtual microscopy imaging) (Belthangady and Royer, 2019; Laine et al., 2021; Lambert and Waters, 2023). For example, a better understanding of how intensity-based quantifications should be performed from virtually enhanced images is very much needed. Biological image data exhibit considerable variability from factors, such as sample physiology, protocols, instrumentation and even individual researchers. Therefore, establishing accurate ‘ground truth’ data is critical (Laine et al., 2021; Maška et al., 2023) given that deep learning model training depends heavily on input data quality. This encompasses factors ranging from the number of training images to their relevance for the intended analytical task. For example, defining the ideal sampling frequency to enable precise cell tracking requires determining the right balance between data acquisition and model performance. Although larger training datasets are thought to enhance model accuracy, strategies to effectively combine diverse datasets, while retaining the specificity of individual experimental conditions, remain to be developed. Publicly available annotated datasets are expanding (Caicedo et al., 2019; Ouyang et al., 2019; Maška et al., 2023), along with pre-trained models to facilitate transfer learning and fine-tuning, such as the Bioimage Model Zoo (Ouyang et al., 2022 preprint) and MONAI (Cardoso et al., 2022). However, best practices for assembling suitable training data and executing productive transfer learning must still be established, considering criteria such as data quality, image traits and analytical goals. Given that live-cell images are highly redundant, such optimization could maximize information extraction, while minimizing photodamage during acquisition.

Unsupervised deep learning approaches learn and match data distributions even in highly heterogeneous or complex scenarios without the need for human descriptions or annotations. Thus, advancing generative models and unsupervised and/or self-supervised approaches that can effectively learn from unpaired data alone can provide flexibility when paired datasets are difficult to obtain experimentally (Box 2) and so contribute to unbiased observations. Moreover, such methods could be exploited to identify the events that deviate from the general distribution, i.e. to discover new biological patterns (Pinkard and Waller, 2022).

Life scientists have extensive expertise in determining optimal parameters, including sampling frequencies, resolution and fields of view for microscopy experiments. Although these hand-tuned parameters might sometimes be suboptimal for subsequent computational analysis and quantification, they currently provide our best reference for what constitutes a ‘high quality’ image and for evaluating when obtained quantifications are accurate. One promising direction is to incorporate user knowledge and experience more directly into the image processing loop to help guide model performance towards more biologically relevant outputs, specific to each experiment. Recent advances, such as creating analytical representations of sparse or raw user inputs to generate priors, offer routes to achieve this. Priors refer to probability distributions that encode assumptions about the data that is going to be analysed. For example, indicating the location of the object to segment with a model. These priors constrain the solutions by reducing the space of possibilities. This general approach has already been proposed for segmenting natural images, such as in the segment anything model (SAM) (Kirillov et al., 2023). Overall, incorporating techniques to integrate human-based feedback as priors into the deep learning pipeline (i.e. the scientist-in-the-loop) represents an important step towards bringing AI-enhanced microscopy closer to matching human experience and intuition.

## Conclusion

Fluorescence microscopy has become an indispensable tool for gaining unparalleled insights into biomolecular dynamics in cell biology. However, phototoxicity remains a major impediment that necessitates both deeper mechanistic understanding and new imaging techniques to mitigate these limitations. Although emerging synergies between microscopy hardware innovation and computational imaging show promise, standardised methodologies to comprehensively assess photodamage are still lacking. Recent advances in deep learning have made progress by enhancing information extraction from low-light or accelerated acquisitions, thereby reducing sample phototoxicity. However, more robust validation strategies are still required to ensure biological fidelity. In order to ensure the success of deep learning-enhanced microscopy, it is crucial to validate it through quantifiable image properties and sample physiology metrics. It is important to benchmark the key image characteristics, such as SNR, resolution limits and molecular content accuracy, against phototoxicity levels. At the same time, it is essential to establish sensitive biological measures that can detect even the slightest deviations from expected cellular behaviour caused by light exposure. Ideally, photodamage assessments should provide actionable and quantitative feedback on imaging protocols, enabling microscopists to optimize the balance between data quality and sample health.

There is a significant opportunity to create universal metrics for photodamage that can account for the incremental effects of light on living samples. Non-invasive techniques such as label-free transmitted light imaging can be used to monitor gradual changes in morphology, metabolism or motility. Additionally, identifying molecular biomarkers of photostress that are accessible through gentle imaging can have a profound impact. It is crucial to have a quantitative damage-reporting system that can detect early warnings, rather than only overt cytotoxicity, and allow for real-time optimization during live acquisition. By incorporating such quantitative damage assessments into intelligent automated analysis workflows, microscopes can dynamically optimise imaging conditions for each specimen. Realising this will require converging advances across several domains: (1) improving biological knowledge of photodamage mechanisms, (2) advancing microscope hardware designs, (3) creating new computational imaging techniques such as deep learning, and (4) accurately interpreting model outputs. A remaining challenge is that deep learning model training requires extensive paired datasets that sufficiently encapsulate the inherent biological variability. Unsupervised learning alternatives provide flexibility but might compromise accuracy compared to supervised techniques. Incorporating biological expertise through techniques such as firstly, priors, which are probability distributions that encode assumptions to constrain solutions, and, secondly, prompts, which provide contextual guidance for generative models, appears promising for guiding model training. Additionally, the field needs empirically driven strategies to optimise model training and validation protocols.

As AI continues to improve imaging capabilities, it is important to remember that the ultimate goal is not just to recover data, but to uncover biological truths. To achieve this, we must prioritise minimising photodamage over pushing technical limits and over relying on computational fixes. Simply relying on technology is not enough, instead the focus should be on maximising information with minimum invasiveness. We must approach innovation with the mindset that it should serve to observe life with minimal perturbance. Therefore, the principles of gentle acquisition and relevant observation of living systems should be the driving force behind future innovations.
